# The driving factors, configuration paths and effects of employment polarization under the development of digital economy

**DOI:** 10.1371/journal.pone.0314362

**Published:** 2024-11-25

**Authors:** Pengfei Zhou, Xianfeng Li, Yang Shen

**Affiliations:** 1 School of Economics and Management, Chongqing Normal University, Chongqing, China; 2 Institute of Quantitative Economics and Statistics, Huaqiao University, Xiamen, China; Sichuan University, CHINA

## Abstract

Taking the development of China’s digital economy as a background, the article provides an in-depth analysis and summarises the influencing factors of labour force employment polarisation. The study employs provincial panel data from 2010 to 2020, applies fuzzy set qualitative comparative analysis methods, and constructs a fixed-effects model in order to explore the drivers, paths and their effects of employment polarisation. The results of the study show that economic, social and educational environments together have a significant impact on employment polarisation through interactions and synergistic effects; it also identifies four main paths of labour force employment polarisation, which are numerical-social-environment-driven, numerical-educational-environment-driven, numerical-economic-environment-driven, and types driven by other factors; this study also finds that compared to the summed impact of single elements, these grouping pathways have a more significant impact on employment polarisation. These findings not only provide a key perspective for understanding how the digital economy shapes employment polarisation, but also provide an empirical basis and insights for policies based on the findings.

## 1 Introduction

The rapid development of intelligence, information technology and digitalisation has led to a profound change in the times. Technological progress has increased the demand for labour skills [1], so that the structure of employment in all countries of the world has begun to change, and the labour market in some countries has begun to appear the phenomenon of job polarisation [2,3]. With the advancement of digital technology and the spread of knowledge, the job market is undergoing profound changes [4]. In this process, the demand for talent in different industries has also shifted. Employment opportunities in traditional manufacturing and agriculture are on a declining trend, while the emerging service and technology industries are facing a growing demand for talent, and these industries are setting higher standards for job seekers’ academic qualifications and professional skills [5]. Currently, structural employment imbalances, regional development differences, and the disconnect between the education system and job market demand are becoming increasingly prominent. In order to address these challenges, the General Office of the State Council of China has officially released the "Notice on Optimizing and Adjusting Policies and Measures to Stabilize Employment and Promote Development and Benefit People’s Livelihoods", which proposes a series of measures aimed at expanding employment scale and broadening employment channels [6], and specifically targets the two major difficulties of "employment difficulties" and "recruitment difficulties", in order to alleviate social employment pressure.

In 2023, the Central Committee of the Communist Party of China (CPC) and the State Council jointly released the Overall Layout Plan for the Construction of Digital China, which marks that the construction of digital China has entered a new stage of rapid development [7]. In this process, digital talents have become the core driving force for the digital transformation of society [8], and the booming development of the digital economy not only heralds a major shift in the global economic landscape, but also has far-reaching impacts on the job market. On the positive side, the digital revolution has injected new vitality into the job market, with the rapid development of emerging industries bringing a large number of new employment opportunities and placing higher demands on the skills of the workforce [9], prompting the labour market to develop in a more knowledge-intensive and innovation-driven direction. However, from another perspective, the wave of digitisation has also triggered structural adjustments in traditional industries, with the development of automation and intelligent technologies putting some low-skilled and repetitive jobs at risk of being replaced [10], leading to a reduction in employment opportunities in traditional manufacturing and service industries, which in turn may lead to the problem of structural unemployment.

By constructing a theoretical framework for the digital economy and employment polarisation, it refines the understanding of the impact of the interaction between the digital economy and other factors on changes in the employment structure, which provides a new theoretical perspective for understanding the impact of the digital economy on the job market. Provides an empirical basis for policy formulation, suggesting the promotion of digital transformation, the improvement of the social security system, the reform of the education system, and the enhancement of the employment competitiveness of high and low-skilled workers, which emphasises the importance of considering a combination of multiple factors in policy formulation to address the challenges posed by employment polarisation. An in-depth study of the impact of the digital economy on different industries and jobs can not only deepen our understanding of the changes in the employment structure, help the government to respond more effectively to potential social challenges, and alleviate the problems of social inequality and unemployment triggered by the changes in the employment structure; it can also promote the sustainable development of the economy, and alleviate the pressures brought about by social inequality and unemployment by adapting the education system to better meet the market demand; in addition, the By analysing the reform of labour market mechanisms in the context of the digital economy, support can be provided for the formulation of more flexible and adaptive labour policies, which are of great practical significance for maintaining long-term social stability.

## 2 Literature review

### 2.1 A study of the impact of the digital economy on employment

The booming development of the digital economy has reshaped the job market on multiple levels [11]. On the one hand, by creating an effect mechanism, it has spawned new employment opportunities, especially in cutting-edge fields such as artificial intelligence, big data, and blockchain technology. The rapid growth of these emerging industries has not only spawned a large number of new jobs, but also driven an urgent demand for professional talents. Meanwhile, with the deepening of digital transformation, the rise of innovative enterprises has invested heavily in technological innovation and other aspects, further bringing abundant employment opportunities to the high-tech field [12]. On the other hand, the compensation effect of the digital economy has significantly improved production efficiency through the widespread use of digital tools [13], which not only promotes the expansion of production scale and the growth of effective demand, but also correspondingly increases the demand for labor. However, the improvement of production efficiency may also reduce labor demand in certain fields through substitution effect mechanisms, leading to a reduction in employment share [14]. Especially, the application of digital technology poses a risk of automation substitution for many traditional, repetitive, and standardized jobs, thereby posing a threat to low skilled labor force unemployment [15].

The impact of the digital economy on employment is complex and multidimensional, and it depends on the dynamic balance between the creation effect, compensation effect and substitution effect [16]. Many scholars believe and the creation effect and the compensation effect are dominant, i.e., the digital economy plays a positive role in the development of the job market [17,18]. On the contrary, if the substitution effect becomes dominant, the digital economy may have a negative impact on employment. If these three effects can reach a dynamic balance, total employment may not be significantly affected, indicating that the overall effect of the digital economy on employment is neutral.

### 2.2 Study of the drivers of the employment structure

In the field of research on labor employment structure, Autor et al. [19] found that the number of moderately skilled positions in the US labor market is gradually decreasing, while the demand for high and low skilled positions is increasing, forming a phenomenon known as "employment polarization". Michaels et al.[20] also pointed out that the development of information and communication technology (ICT) can lead to polarization in the labor market. In China, there are differences in discussions regarding changes in the employment structure of the labor force. Some scholars believe that, similar to Western countries, China’s employment structure is also showing a polarization trend. The study by Liu and Lu [21] pointed out that the improvement of the development level of new factor kinetic energy will lead to polarization of the labor market, which is reflected at the micro level in the increase or decrease of conventional and unconventional tasks. Sun and Hou [22] emphasized the substitution effect of industrial intelligence on the labor force with junior high and senior high school education, believing that this will lead to polarization of the employment structure. However, there are also views that the employment structure of China’s labor force is mainly upgrading. The study by Hui and Shan [23] found that the employment structure is influenced by multiple factors with industrial intelligence as the core, which together promote the upgrading of the labor force skill structure. Currently, most researchers agree that the development of digitalization has played a promoting role in the employment of highly skilled labor force [24,25]. When exploring the mechanism of this phenomenon, scholars have analyzed it from different perspectives. Cong and Yu [26] empirically tested how artificial intelligence promotes the upgrading of labor employment structure through technological innovation effects and talent agglomeration effects from an innovation driven perspective, and further pointed out that the impact of artificial intelligence on employment structure has non-linear characteristics, especially in developed coastal areas where this effect is more significant. Yang et al. [27] used labor productivity in various industries as an indicator to measure technological progress and found that digital investment affects employment structure by promoting technological progress. In addition to these factors, industrial structure upgrading [28], cognitive abilities [29], and skill premiums [30] are also important factors driving changes in employment structure.

### 2.3 Literature review and innovations

The theoretical framework has matured in the field of research exploring the impact of the digital economy on employment structure. The existing literature generally focuses on the substitution effect of AI technology on the employment structure and the single-path mechanism. At the same time, research has also begun to focus on the mediating role of industrial structure upgrading, technological innovation and technological progress in the change of employment structure. However, the reality shows that it is difficult for a single factor to independently drive structural changes in the labour market. The evolution of the employment structure of the labour force is a complex process that is affected by a combination of factors. When analysing changes in employment structure, current research often fails to fully consider the complexity of the interaction of multiple factors, which limits the depth and breadth of the theory to a certain extent. Future research should take multiple factors into account in order to reveal more comprehensively the internal mechanism of employment structure change in the context of digital economy. In the study of the role of the digital economy in influencing the employment structure, the theoretical mechanism has been relatively perfect. Most of the driving factors of the employment structure focus on the substitution role of artificial intelligence and the mechanism of a single path, taking into account the intermediary role of industrial structure upgrading, technological innovation and technological progress and other factors. However, in reality, it is difficult for a single factor to change the structure of the labour force employment market independently, and changes in the employment structure of the labour force are affected by a variety of reasons, and existing research lacks the consideration of the impact of multiple factors on the employment structure. The innovation of this study is mainly reflected in three aspects:

1. At the theoretical level, the article systematically considers the factors that affect the employment structure, providing a more comprehensive perspective on the impact mechanism of the labor market and providing more precise and flexible policy recommendations for policymakers.
2. Secondly, in terms of methodology, this article adopts the configuration path analysis method to explore the influencing factors of employment structure. Due to the fact that the formation of employment structure is the result of multiple factors working together, many of which are difficult to quantify or have ambiguity. Fuzzy set qualitative comparative analysis can effectively handle these uncertainties, while considering the complex interactions between elements, comprehensively evaluating multidimensional factors to draw more comprehensive conclusions.
3. Thirdly, from a contingency perspective, this article compares the cumulative effects of single factor influence with the impact of configuration factors on employment structure, thereby verifying the advantages and effectiveness of configuration path analysis in revealing the influencing factors of employment structure.

## 3 Design of the research framework and description of variables

### 3.1 Theoretical description

#### 3.1.1 Digital economy development

The development of digital economy is reshaping the labor force employment structure with three mechanisms: creation effect, compensation effect and substitution effect [31]. With the development of the digital economy, the supply and demand of the labor force is experiencing significant changes, and the transformation of occupations and industries is taking place in parallel [32]. For example, the manufacturing industry is accelerating towards automation, while the traditional retail industry is subject to fierce competition from e-commerce marketplaces. These transformations have led to a decrease in the demand for labor in traditional industries, while at the same time, the demand for jobs in emerging fields such as data analytics and software development has been rising, which in turn has pushed up the demand for high-skilled labor, while the supply of low-skilled labor has been relatively reduced, which has had a far-reaching impact on the employment structure of the workforce. In addition, the development of the digital economy has driven technological progress, and technological innovation has changed the mode of production and the combination of factors of production, resulting in the gradual replacement of traditional labor-intensive jobs by machines and artificial intelligence. At the same time, the widespread application of digital technologies requires more highly skilled personnel with technical, managerial and innovative capabilities to develop, maintain and manage related systems [33], which further changes the labor force employment structure. Ultimately, the development of the digital economy has enhanced the flexibility of the market mechanism, with the rise of digital economic platforms giving rise to a large number of odd-job economic work models, and the spread of teleworking and digital communication technologies providing workers with more diversified work options. These adjustments in market mechanisms have also had an impact on the employment structure of the workforce, making it more diverse and complex.

Technological progress in China exhibits a dual bias of capital and skills. According to the skill biased theory of technological progress, the development of technology increases the marginal output of highly skilled labor [34], leading to an increase in their relative wage levels, which increases the demand for high skilled and knowledge intensive jobs. In addition, some scholars have proposed the concept of conventional biased technological progress [35], which provides a detailed classification of work tasks, pointing out that technological progress encourages highly skilled workers to engage more in unconventional intellectual labor tasks, while low skilled workers tend to shift more towards unconventional manual labor tasks. Technology-labour substitution theory points out that with technological progress, enterprises are more inclined to use machines and equipment to replace labor, which leads to a decrease in demand for low skilled jobs, while medium skilled labor with high job content repetition is more easily replaced by intelligent assembly lines. This theory explains the phenomenon of a decrease in demand for medium skilled labor [36]. The impact of technological progress on employment structure is influenced by various factors such as economic conditions, talent structure, education and training, social security, and employment policies. Therefore, when analyzing the evolution of labor employment structure and its influencing factors, it is necessary to systematically classify and conduct in-depth analysis of these factors. This classification should start from multiple dimensions and comprehensively evaluate the impact of various factors on the employment structure of the labor force.

Institutional theory emphasises the influence of the institutional environment on economic behaviour [37]. Institutional factors in the economic, educational and social environments combine to shape the pattern of employment polarisation. According to political economy, the economic environment includes factors such as market structure and property rights, which influence production methods and labour demand, and thus employment polarisation. Educational Expansion theory discusses the impact of the spread of education on the labour market, where the expansion of the educational environment changes the skill structure of the workforce [38]. Social stratification theory explores how social structures and institutions lead to unequal access to resources for members of society, affecting individual opportunities in the labour market [39]. Considering the hierarchical structure of labor relations, systematic thinking should be conducted on three factors: economy, society, and education in order to comprehensively consider the impact of different environments on the labor market and avoid analysis that is too scattered and isolated [40]. This not only helps the government and relevant institutions to formulate more targeted employment promotion policies and measures based on these three key elements, but also provides theoretical basis for precise policy formulation. Based on the development background of the digital economy, this article will explore in detail how these factors affect the path and degree of polarization of labor employment in China. The specific factors are as follows:

#### 3.1.2 Economic environment

The development of industries and industries has created the most direct economic environment for employment [41]. This article mainly analyzes the impact of economic environment on employment polarization from two aspects: industrial structure upgrading and industrial agglomeration. One is the upgrading of industrial structure, which has a significant impact on employment structure. With the evolution of industrial structure, the relative importance of different industrial sectors and their requirements for skills are also adjusted accordingly. Skill-biased technological advances have led to changes in the demand for labor and the distribution of employment opportunities, thus contributing to a shift in the shape of the employment structure. The second is industrial agglomeration, where population concentration not only promotes industrial clustering development [42], but also, according to the hierarchy of demand theory, the knowledge spillover effect brought by population agglomeration increases the proportion of medium to high skilled labor force [43], which further leads to the evolution of labor force structure.

#### 3.1.3 Social environment

The social environment has a significant impact on the changes in employment structure, which is reflected in the integration of social structure and institutions. The article selects three dimensions: population aging, government intervention, and urbanization level to analyze the impact of social environment on employment polarization. One is population aging, which through demand effects, productivity effects, and backforcing mechanisms [44], promotes enterprises to accelerate transformation and reform, change production methods, and thus promote the reshaping of labor employment structure. The second is government intervention, which plays a complex role in the changes in employment structure. On the one hand, if the welfare and subsidies provided by the government are too high, it may lead to low skilled labor choosing not to work due to government subsidies exceeding the wage level; On the other hand, government training programs and entrepreneurship subsidies can enhance the skill level of workers, thereby promoting the polarization of the high skilled labor market [45]. The third is the level of urbanization. From the perspective of spatial externalities, urbanization not only promotes the sharing of information and resources, but also accelerates the spillover of knowledge [46]. These factors have led to changes in the geographical distribution of employment opportunities, increased occupational diversity, and upgraded skill demands, further driving the evolution of the labor force employment structure [47].

#### 3.1.4 Educational environment

The improvement of the education system is key to meeting the labor market’s demand for different skill levels [48]. Workers who have received higher education usually have stronger cognitive and learning abilities, can quickly adapt to the reform needs of enterprises, and thus be competent for more unconventional intellectual work tasks [49]. This element takes into account the factors influencing employment in the labor market from a supply side perspective, thereby promoting the transformation of the labor structure. Therefore, further analyzing the impact of education environment on employment polarization through two key indicators: human capital level and education expenditure. One is the level of human capital, and the improvement of human capital level means that there are more workers with advanced skills and profound educational background in the job market [50]. Workers have upgraded their skills through learning and training, enabling them to handle more complex job responsibilities and thereby changing their employment structure. The second is education expenditure, and the increase in education expenditure is a guarantee for improving the quality of education. A high-quality education system can cultivate a large number of high-quality labor force, which not only promotes the transformation of labor force from low skilled occupations to high skilled occupations, but also has an impact on the employment structure of labor force.

### 3.2 Research framework

**Fig 1:**
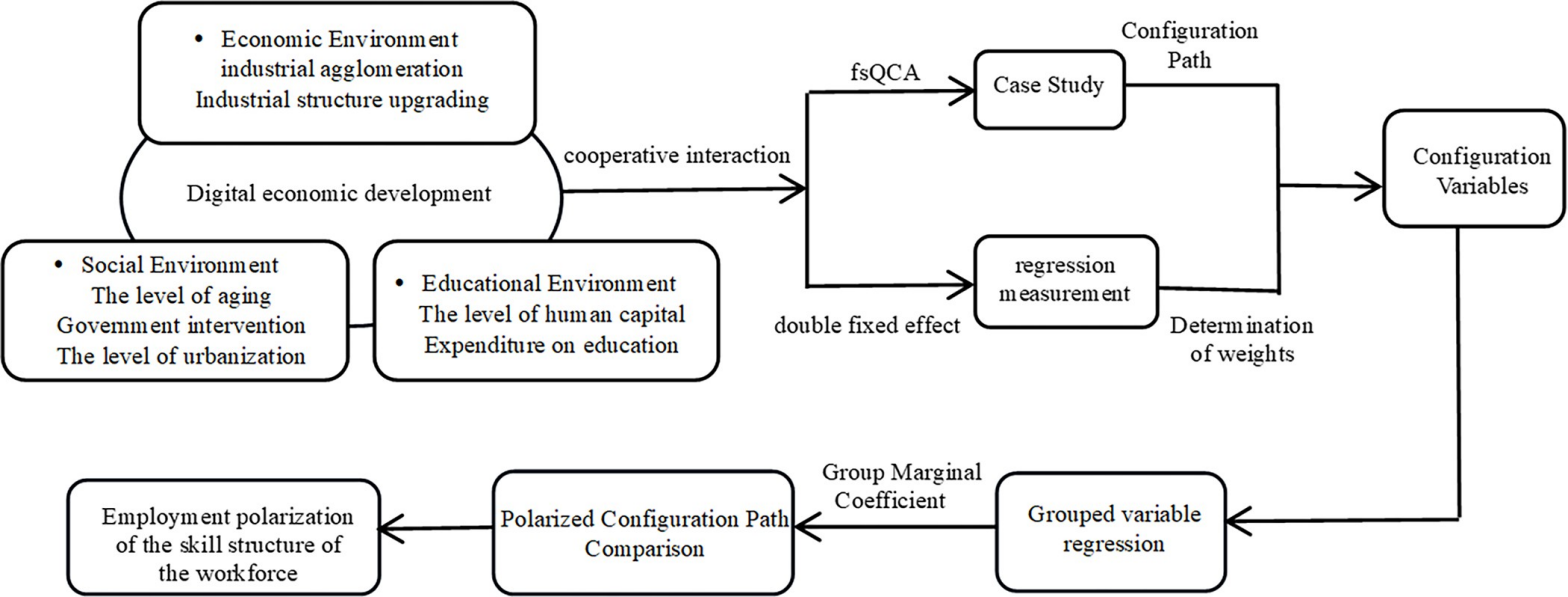
Design of the research framework.

Through the analysis above, we realize that in the context of rapid development of the digital economy, the phenomenon of employment polarization is a comprehensive result of the interaction of economic, social, and educational environment factors. Based on this, this study sets these factors as conditional variables and the polarization of labor employment structure as outcome variables. Based on a configuration perspective, explore how these factors work together and affect employment polarization, and use econometric analysis to determine the magnitude of the net impact of individual factors. Considering that the regression coefficient can reflect the importance of the independent variable to the dependent variable, the regression coefficient is used as a weight to linearly weight the configuration variable, in order to further determine the marginal effects of the configuration path through regression analysis. By comparing the total net impact of various factors with the marginal effects of configuration variables, we can evaluate the specific impact of different configuration paths on labor employment polarization. The research framework is shown in Fig 1.

### 3.3 Variable selection

#### 3.3.1 Result variable

Employment polarisation (EMP): The former academic classification of labour force skills does not form a unified standard, the article uses the educational level of labour force personnel to measure the level of skills. The education level below junior high school is classified as low-skilled labour force [51], the education level of bachelor degree and above is classified as high-skilled labour force [52], and the rest of the education level is medium-skilled labour force, and the employment polarization is expressed by the ratio of high and low-skilled labour force to medium-skilled labour force.

#### 3.3.2 Conditional variable

(1) Digital economic development (DED): The article evaluates the level of digital economic development based on the China Digital Economic Development Index jointly released by CCID Consulting and Deyang, Sichuan. Combining existing achievements, it selects four dimensions: digital foundation [53], digital application, digital innovation [54,55], and digital benefits. 21 indicators are used to evaluate the level of digital economic development. The specific indicator system is shown in Table 1. The measurement method for the digital economy adopts the objective entropy weight method to determine the weights of each indicator and calculate the level of digital economy development (DED) [56]. Due to space limitations, the specific calculation process is not listed here.

**Table 1:** Digital economy development measures.

First indexes	Second indexes	Indicator properties
**Digitization Foundation**	Length of fiber optic cable	+
	Number of cell phone base stations	+
	Cell phone penetration rate	+
	Number of Internet broadband access ports	+
**Digitization Applications**	Number of people with Internet access	+
	Number of Internet domain names	+
	Software industry revenue	+
	Breadth of digital financial coverage	+
	Depth of use of digital finance	+
	Degree of digital finance digitization	+
	Level of online mobile payments	+
**Digitization Innovation**	Full-time equivalents of R&D personnel in industrial enterprises above large scale	+
	R&D Expenditures of Industrial Enterprises Above Scale	+
	Number of R&D projects (topics) of industrial enterprises above large scale	+
	Total technology contract turnover	+
	Number of patent applications	+
	Number of patent applications for inventions	+
	Number of patent applications granted	+
**Digitization benefits**	Number of employees in the information services industry	+
	Output value of information services	+
	Volume of telecommunication services	+

(2) Industrial structure upgrading (ISP): represented by the industrial upgrading structure coefficient [57].


$$ISP=\sum_{i=1}^{3}y_{i}\times i=y_{1}\times 1+y_{2}\times 2+y_{3}\times 3\;1\leq R\leq 3$$ (1)

In Eq (1), *y*_*i*_ represents the proportion of the added value of the i-industry to the total output value, with a value range of R ranging from 1 to 3. The closer R is to 1, the lower the level or height of industrial structure, indicating that the economy and society belong to agricultural society; If R is close to 2, it indicates that the level or height of industrial structure is moderate, and the economy and society belong to industrial society; The closer R is to 3, the higher the level or height of the industrial structure, indicating that the economy and society belong to the post industrial information and knowledge society; (3)Industrial agglomeration (IA) is expressed as the ratio of the number of employed persons to the area of the administrative division; (4) The level of aging (TLA) is expressed in terms of the dependency ratio of the aging population in each province; (5) Government intervention (GI) is measured by the ratio of fiscal expenditure on social security and employment to GDP in each province; (6) The level of urbanization(TLU) is measured by the proportion of the urban population to the total population; (7) The level of human capital (LHC) is expressed as the ratio of the number of students enrolled in higher education schools to the total population; (8) Expenditure on education (EOE) is expressed as the ratio of the state financial expenditure on education to the general budgetary expenditure.

### 3.4 Data sources

The article adopts panel data analysis from 2010 to 2020, which is sourced from various years such as the China Labor Statistics Yearbook, China Population and Employment Statistics Yearbook, China Statistical Yearbook, China Third Industry Statistics Yearbook, China Information Industry Yearbook, as well as the Peking University Digital Inclusive Finance Index, National Bureau of Statistics, and provincial statistical yearbooks. In addition to Xizang, where there are many missing values, some missing values are supplemented by linear interpolation. Table 2 reports the descriptive statistical results of the corresponding variables.

**Table 2:** Descriptive Statistics.

Variable name	Code	Obs	Mean	Standard	Min	Max
Employment polarization	EMP	300	0.207	0.053	0.082	0.360
Digital economic development	DED	300	0.122	0.112	0.010	0.752
Industrial structure upgrading	ISP	300	2.374	0.129	2.166	2.836
Industrial agglomeration	IA	300	0.026	0.038	0.000	0.217
The level of aging	TLA	300	12.803	3.475	5.390	24.700
Government intervention	GI	300	0.250	0.103	0.110	0.643
The level of urbanization	TLU	300	0.590	0.122	0.350	0.896
The level of human capital	LHC	300	0.020	0.005	0.008	0.041
Expenditure on education	EOE	300	1.248	1.775	0.031	10.488

## 4 Grouping path analysis for employment polarization

### 4.1 Research methodology

Qualitative comparative analysis (QCA) is a qualitative comparative analysis method based on set theory and Boolean algebra. It is case oriented and adopts a "configuration" approach to analyze and process a limited number of complex cases. Provide a systematic comparative analysis method that believes that the outcome variable cannot be independently triggered by a single factor, but rather has multiple concurrent causal relationships. Fuzzy set qualitative comparative analysis (fsQCA) requires a strong mathematical foundation in the QCA method [58]. It uses the fuzzy logic method in fuzzy set theory to transform factors and outcome variables into fuzzy sets, and uses fuzzy set operators to analyze and derive causal or propensity relationships. FsQCA focuses on the relationship between exclusivity and inclusivity, and does not require strict linear relationships. Not only does it integrate the strengths of both qualitative (case oriented) and quantitative (variable oriented) analysis methods, but it can also handle partial membership problems in sets.

### 4.2 Calibration of data

Data calibration is a necessary step in conducting fsQCA analysis, and this article calibrates the antecedent and outcome variables through direct calibration. Drawing on the published literature, the 95%, 50%, and 10% percentile values of the variables were used as calibration anchors for complete membership, intersection, and complete non membership [59]. The specific calibration values are shown in Table 3.

**Table 3:** Calibration of data.

Variable name	Fully affiliated	Intersections	Not affiliated
**Employment polarization**	0.355	0.081	0.035
**Digital economic development**	2.674	2.361	2.237
**Industrial structure upgrading**	0.075	0.015	0.003
**Industrial agglomeration**	19.316	12.355	8.795
**The level of aging**	0.436	0.226	0.137
**Government intervention**	0.867	0.574	0.451
**The level of urbanization**	0.029	0.019	0.014
**The level of human capital**	2.619	0.757	0.118
**Expenditure on education**	0.304	0.209	0.141

### 4.3 Necessity analysis of antecedent conditions

Following the fsQCA research standard, it is necessary to analyze the necessity of a single conditional variable (including its non set) to test whether a factor is a necessary condition for the outcome variable. If a antecedent variable always exists when the outcome variable occurs, then that antecedent condition is a necessary condition for the outcome variable. Consistency refers to the degree of consistency between the conditional variable and the outcome variable, which tests whether the set of outcome variables is a subset of the antecedent conditions. The baseline for consistency is 0.9. When consistency exceeds 0.9, the antecedent condition is considered a necessary condition for the result. The necessary conditions for employment polarization are shown in Table 4, and the consistency of all antecedent conditions is less than 0.9, so there is no necessary condition for employment polarization in the antecedent conditions. The coverage of antecedent conditions is between 0.4806 and 0.8017, indicating that they have a certain explanatory power for employment polarization.

**Table 4:** Necessity analysis.

Antecedent condition	consistency	Coverage
**Digital economic development (**DED**)**	0.7034	0.7403
**~ Digital economic development (~ **DED**)**	0.5261	0.4825
**Industrial structure upgrading (**ISP**)**	0.7070	0.7225
**~ Industrial structure upgrading (~ **ISP**)**	0.5298	0.4989
**Industrial agglomeration (**IA**)**	0.7193	0.8017
**~ Industrial agglomeration (~**IA**)**	0.5521	0.4829
**The level of aging (**TLA**)**	0.6323	0.6621
**~ The level of aging (~**TLA**)**	0.6094	0.5613
**Government intervention (**GI**)**	0.4833	0.4806
**~ Government intervention (~**GI**)**	0.7595	0.7338
**The level of urbanization (**TLU**)**	0.7321	0.7382
**~ The level of urbanization (~**TLU**)**	0.5316	0.5068
**The level of human capital (**LHC**)**	0.7324	0.7330
**~ The level of human capital (~**LHC**)**	0.5483	0.5266
**Expenditure on education (**EOE**)**	0.7285	0.7471
**~ Expenditure on education (~**EOE**)**	0.5130	0.4814

### 4.4 Adequacy analysis of conditional configuration

From the perspective of set theory, configuration analysis aims to explore whether a specific conditional configuration composed of multiple antecedent conditions constitutes a subset of the set of outcome variables, and evaluate the adequacy of these conditional configurations for the outcome variables. Drawing on the experience of Du [60], the parameters were first set, with a consistency threshold of 0.85, a case frequency of 1, and a PRI consistency threshold of 0.7. Based on this, a truth table was constructed. Then, based on the nested relationship between the intermediate solution and the simplified solution obtained, the core condition and edge condition are determined. The condition that both the intermediate solution and the simplified solution appear simultaneously is defined as the core condition, and the condition that the intermediate solution exists but the simplified solution does not appear is defined as the edge condition. The obtained employment polarization condition configuration is shown in Table 5. Overall, there are four configuration paths that cause employment polarization, with consistency of 0.9027, 0.8973, 0.9524, and 0.9675, respectively. The overall consistency is 0.9008, indicating that these paths can all be considered sufficient conditions for employment polarization. The overall coverage is greater than 0.5, indicating that each condition configuration has strong explanatory power for the formation of employment polarization.

**Table 5:** Configuration path.

Antecedent condition	Employment polarization			
	H1	H2	H3	H4
**DED**	●	●	●	
**ISP**	●	●	●	●
**IA**	●	●	●	●
**TLA**		●	⊗	⊗
**GI**	⊗	⊗		⊗
**TLU**	●		●	●
**LHC**		●	●	●
**EOE**	●	●	●	●
**Consistency**	0.9027	0.8973	0.9524	0.9675
**Raw coverage**	0.4473	0.3352	0.2697	0.2832
**Unique coverage**	0.0746	0.0154	0.0172	0.0307
**Solution Consistency**	0.9008			
**Solution coverage**	0.5106			

Note(s): ●Indicates the existence of core conditions, ●Indicates the existence of edge conditions, ⊗ Indicates that the core condition does not exist,⊗ Indicates the absence of marginal conditions.

### 4.5 Analysis of the configuration path of labor force structure

There are four paths for employment structure polarization, among which industrial structure upgrading is the core condition and a key factor in the formation of employment polarization. There are edge conditions for the development of H1 configuration digital economy, and two core variables, government intervention and urbanization level, exist in the social environment. Therefore, this path is named digital social environment driven; The H2 configuration of digital economy development has marginal conditions, and the conditional variables of education environment, such as human capital level and core conditions of education expenditure, exist. Therefore, it is named as a digital education environment driven model; The H3 configuration has the edge conditions for the development of digital economy and the core conditions for industrial agglomeration, and it is named as a digital economy environment driven model; Finally, the lack of conditions for the development of the H4 digital economy is referred to as driven by other factors. Below is an analysis of these four paths and their corresponding typical cases.

#### 4.5.1 Digital social environment driven

The conditional configuration of this path is: DED * ISP * IA *~GI * TLU * EOE (digital economy development * industrial structure upgrading * industrial agglomeration *~government intervention * the level of urbanization * expenditure on education). Under this path, when the regional industrial structure upgrading, urbanization level, and education expenditure are all at a high level, while the digital economy development and industrial agglomeration remain at a relatively high level, and the degree of government intervention is low, the phenomenon of employment structure polarization will occur. In addition, the impact of population aging and human capital level on the degree of polarization in this pathway is not significant. The upgrading of industrial structure is usually accompanied by technological progress and changes in production methods. The development of the digital economy and industrial agglomeration have created more employment opportunities for the service industry and high-tech fields. The advancement of urbanization has led to a greater concentration of employment opportunities, while high-level education expenditures ensure the matching of labor skills with market demand. A low level of government intervention means that the market can operate more freely, which improves the match between technology and knowledge intensive positions and high skilled, highly educated talents. However, the concentration of high-level talent may lead to downward mobility of medium-skilled labor, exacerbating the trend of dualization in the job market. This path can explain 45% of employment polarization cases, with Guangdong and Zhejiang provinces being typical representatives. Guangdong and Zhejiang actively promote digital transformation and industrial upgrading, forming an industrial agglomeration effect and injecting new impetus into economic development. The urbanization rate of the two provinces far exceeds the national average, attracting a large number of talents, but it also brings about the phenomenon of uneven employment. The full utilization of market mechanisms and the emphasis on independent decision-making by enterprises have created a large number of high paying and skilled employment opportunities. However, medium skilled workers who cannot adapt to intelligent technology may be forced to shift towards low skilled jobs, such as personal care and health services, which are difficult to replace with artificial intelligence, thereby exacerbating the polarization of the job market.

#### 4.5.2 Digital education environment driven

The configuration of this path condition is: DED * ISP * IA * TLA *~GI * LHC * EOE (digital economy development * industrial structure upgrading * industrial agglomeration * the level of aging *~government intervention * the level of human capital * expenditure on education), revealing that in the context of upgraded industrial structure and high levels of both human capital and education expenditures, employment polarization is likely to occur even if the level of urbanization is low, as long as the development of the digital economy, industrial agglomeration, and population aging are maintained at a relatively high level and the level of government intervention is low. Under these conditions, high skilled talents enjoy more employment opportunities in areas with developed digital economy and industrial structure. The decrease in labor supply caused by aging population has intensified the tension and competition in the job market. At the same time, the improvement of the education system and the high level of human capital have enhanced the competitiveness of workers who receive high-quality education, while low skilled workers face the challenge of finding stable, high paying jobs. The lack of government intervention in the job market may further exacerbate polarization. This configuration can explain 33.5% of polarization cases, taking Jiangsu as an example. Jiangsu has a developed industrial structure and, driven by the digital economy, has nurtured a group of globally competitive digital economy enterprises, attracting a large number of highly skilled talents. The province has demonstrated strong strength in terms of educational resources and quality, with significant labor force training and delivery capabilities. However, with the increase of high skilled talents, medium skilled workers are at a disadvantage in competition and may shift to low skilled positions, leading to polarization of the job market.

#### 4.5.3 Digital economic environment driven

The conditional configuration of this path is: DED * ISP * IA *~ TLA * TLU * LHC * EOE (digital economy development * industrial structure upgrading * industrial agglomeration * the level of aging * the level of urbanization * The level of human capital * expenditure on education). The H3 path points out that in the case of high levels of industrial structure upgrading and industrial agglomeration, with the relatively high level development of digital economy, urbanization, human capital, and education expenditure, non aging areas are more likely to experience polarization of labor employment structure, and government intervention is less critical in this path. The vigorous development of the digital economy has driven the industrial innovation in the field of digital technology and the Internet. The rise of emerging industries has not only promoted the upgrading of the industrial structure, but also formed industrial clusters through the cluster effect. These regions have created a large number of employment opportunities with high skills and high salaries, thus increasing the demand for highly skilled labor. With the improvement of education and human capital levels, the labor supply in non aging areas has relatively decreased, the proportion of young labor has increased, and the number of low skilled labor has relatively decreased. In this context, the shortage of high skilled labor and the employment difficulties of low skilled labor have jointly exacerbated the polarization of the employment market. This path can explain 27% of polarization cases, representing provinces such as Beijing and Shanghai. Both cities have established numerous science and technology parks and economic development zones, actively promoting the upgrading and transformation of digital industries. As the economic center of the country, they attract a large number of highly skilled talents. In terms of educational resource allocation, Beijing and Shanghai have also shown advantages, with skill oriented technological progress improving the specialization and technical level of the labor force. Meanwhile, changes in population structure have led to an increase in low skilled and low paying job positions, further promoting the formation of employment polarization.

#### 4.5.4 Other factor driven

The configuration of this path condition is: ISP * IA *~ TLA *~GI * TLU * LHC * EOE (industrial structure upgrading * industrial agglomeration *~ the level of aging *~government intervention * the level of urbanization * The level of human capital * expenditure on education). This indicates that a region’s industrial structure upgrading and education expenditure are both at a high level, with relatively high levels of industrial agglomeration, urbanization, and human capital, and less government intervention. In the case of low population aging degree, it will also lead to employment polarization. The demand for high skilled labor in regions with advanced industrial development and high levels of marketization continues to increase. The overall quality and skill level of the labor force have improved, leading to an increase in the matching degree between a large number of workers and job demands. The loss of high childbearing age labor force may lead to a relative concentration of low skilled labor in the region, resulting in a greater demand for low skilled jobs, leading to employment polarization. H4 configuration can explain 28% of polarization cases, taking Tianjin as an example. As an economic center and important port city, Tianjin has always been committed to developing high value-added industries such as high-tech manufacturing, aerospace, and biopharmaceuticals. These industries have a great demand for highly skilled labor. Although the education environment in Tianjin is relatively favorable, due to less government intervention in the market and a low degree of aging population, the supply speed of high skilled talents cannot keep up with the growth of demand, resulting in a mismatch between supply and demand. In addition, the concentration of economic activities in Tianjin has led to a greater concentration of high skilled and low skilled job opportunities, further exacerbating the polarization of the job market.

### 4.6 Robustness test

There are multiple robustness testing methods in fsQCA, and the most commonly used one is to adjust relevant parameters, including calibration threshold, case frequency, and consistency threshold value. It can also be achieved by adding conditions, supplementing or removing cases. This article increases the frequency of cases from 1 to 2 and lowers the consistency threshold from 0.85 to 0.8 to test the robustness of the configuration path of labor employment polarization. The generated configuration paths H1 and H2 each have a core condition present turned into an edge condition present. H3 and H4 are completely consistent, and each path has a clear subset relationship (Table 6), indicating that the obtained configuration research results are robust.

**Table 6:** Robustness test of configuration paths.

Antecedent condition	Employment polarization			
	H1	H2	H3	H4
**DED**	●	●	●	
**ISP**	●	●	●	●
**IA**	●	●	●	●
**TLA**		●	⊗	⊗
**GI**	⊗	⊗		⊗
**TLU**	●		●	●
**LHC**		●	●	●
**EOE**	●	●	●	●
**Consistency**	0.9027	0.8973	0.9524	0.9675
**Raw coverage**	0.4473	0.3352	0.2697	0.2832
**Unique coverage**	0.0746	0.0154	0.0172	0.0307
**Solution Consistency**	0.9008			
**Solution coverage**	0.5106			

Note(s):●Indicates the existence of core conditions, ●Indicates the existence of edge conditions, ⊗ Indicates that the core condition does not exist,⊗ Indicates the absence of marginal conditions.

## 5 Analysis of the net impact and configuration path impact of individual factors on employment polarization

### 5.1 Model building

To identify the impact of individual factors on employment polarization and determine the marginal utility of variables. Select a fixed effects model based on Hausman’s results. The basic econometric model is set as follows:

$${EMP}_{it}=\beta_{0}+\beta_{1}{DED}_{it}+\beta_{2}{ISP}_{it}+\beta_{3}{IA}_{it}+\beta_{4}{TLA}_{it}+\beta_{5}{GI}_{it}+\beta_{6}{TLU}_{it}+\beta_{7}{LHC}_{it}+\beta_{8}{EOE}_{it}+u_{i}+v_{t}+\varepsilon_{it}$$ (2)

In Eq (2), *i* represents the province and *t* represents the year; The dependent variable EMP_it_ represents the polarization of labor employment structure in province i in year t, DED represents the level of digital economy development, ISP represents industrial structure upgrading, IA represents industrial agglomeration, TLA represents the level of aging, GI represents government intervention, TLU represents the level of urbanization, LHC represents human capital level, and EOE represents expenditure on education. In addition, β is the parameter to be estimated, *u*_*i*_ is an individual factor, *v*_*i*_ is a time factor, and *ε*_*it*_ is a random interference term.

According to fsQCA, four employment polarization configuration paths were obtained, and the relationship between factor configuration and outcome variables was analyzed through case studies in the context of set theory. The coverage of the configuration path calculated by QCA is the explanatory power of the case, but it cannot show the magnitude of the influence of factor variables. Therefore, further use econometric models to calculate the marginal utility of the factors influencing employment polarization and the configuration path, and thoroughly examine the configuration results of employment polarization. The formula is set as follows:

$$E{MP}_{it}=\alpha_{0}+\alpha_{1}{HDS}_{it}+\alpha_{2}{TLA}_{it}+\alpha_{3}{LHC}_{it}+u_{i}+v_{t}+\eta_{it}$$ (3)

$$E{MP}_{it}=\delta_{0}+\delta_{1}{HDE}_{it}+\delta_{2}{TLU}_{it}+u_{i}+v_{t}+\epsilon_{it}$$ (4)

$$E{MP}_{it}=\gamma_{0}+\gamma_{1}{HDC}_{it}+\gamma_{2}{GI}_{it}{+u}_{i}+v_{t}+\omega_{it}$$ (5)

$$E{MP}_{it}=\varphi_{0}+\varphi_{1}{HOF}_{it}+\varphi_{2}{DED}_{it}{+u}_{i}+v_{t}+\theta_{it}$$ (6)

In Eqs (3) to (6), EMP, TLA, LHC, TLU, GI, DED, *u*_*i*_, *v*_*t*_, i and t have the same meanings as Eq (2). α, δ, γ, and φ are the parameters to be estimated, and *η*_*it*_, *ϵ*_*it*_, *ω*_*it*_, and *θ*_*it*_ are random disturbance terms. The econometric models in Eqs (3) to (6) are used for the estimation of the marginal effects of the four grouped variables. HDS, HDE, HDC, and HOF denote the linear combinations of the conditional variables included in the paths of digital social environment-driven, digital educational environment-driven, digital economic environment-driven, and other factors-driven, respectively, weighted by the weights of the regression coefficients in model (2). Namely:

$$HDS=\lambda_{1}DED+\lambda_{2}ISP+\lambda_{3}IA+\lambda_{5}GI+\lambda_{6}TLU+\lambda_{8}EOE$$ (7)

$$HDE=\lambda_{1}DED+\lambda_{2}ISP+\lambda_{3}IA+\lambda_{4}TLA+\lambda_{5}GI+\lambda_{7}LHC+\lambda_{8}EOE$$ (8)

$$HDC=\lambda_{1}DED+\lambda_{2}ISP+\lambda_{3}IA+\lambda_{4}TLA+\lambda_{6}TLU+\lambda_{7}LHC+\lambda_{8}EOE$$ (9)

$$HOF=\lambda_{2}ISP+\lambda_{3}IA+\lambda_{4}TLA+\lambda_{5}GI+\lambda_{6}TLU+\lambda_{7}LHC+\lambda_{8}EOE$$ (10)

In Eqs (7) to (10), λ_k_ is the ratio of the regression coefficient of the kth explanatory variable in Eq (2) to the sum of the regression coefficients of all explanatory variables. The regression coefficient represents the average impact of unit changes in each explanatory variable on the dependent variable, reflecting the magnitude of the impact of the explanatory variable on the dependent variable. It is a natural weight and has been used in literature^]^ to determine the weight for problem analysis[61,62].

### 5.2 Analysis of single factor net impact and configuration path impact on employment polarization

As shown in Table 7, the results of model (2) show that overall, the estimated coefficients of digital economy development, the level of urbanization, The level of human capital, and education expenditure are all positive and pass the test at a significance level of at least 5%. This indicates that when acting alone, these factors may exacerbate the trend of employment polarization. At the same time, the estimated coefficient of industrial agglomeration is significantly negative at the 1% significance level, indicating that excessive industrial dispersion may also lead to employment polarization. However, other variables in the model did not show a significant impact on the skill structure of the labor force statistically. H1 to H4 represent the regression results of different configuration paths, all of which indicate that the corresponding configuration variables exacerbate the polarization of employment structure. Specifically, at a significant level of 1%, for every 1% increase in the digital social environment combination variable, the average increase in employment polarization effect is 3.22%; For every 1% increase in the digital education environment combination variable, the average increase in employment polarization effect is 3.221%; For every 1% increase in the digital economic environment combination variable, the average increase in employment polarization effect is 3.22%; For every 1% increase in other factor combination variables, the average increase in employment polarization effect is 3.22%. The net impact sum of variables included in HDS, HDE, HDC, and HOF under four configuration paths is -1.92, 2.76, 3.21, and 3.15, respectively, which is less than the marginal impact values of 3.22, 3.221, 3.22, and 3.22 for the four configuration variables. This finding suggests that, compared to the net impact of individual variables, the combined variables of configuration paths have a greater marginal impact in inducing employment polarization.

**Table 7:** Regression results.

Variable	Base model	H1	H2	H3	H4
DED	0.073**				0.073***
	(0.031)				(0.028)
ISP	0.031				
	(0.040)				
IA	-2.560***				
	(0.925)				
TLA	0.046	0.046			
	(0.062)	(0.061)			
GI	0.014			0.014	
	(0.051)			(0.046)	
TLU	0.458***		0.458***		
	(0.090)		(0.076)		
LHC	5.119***	5.119***			
	(1.376)	(0.948)			
EOE	0.039**				
	(0.017)				
HDS		3.220***			
		(0.467)			
HDE			3.221***		
			(0.688)		
HDC				3.220***	
				(0.321)	
HOF					3.220***
					(0.327)
Individual	Yes	Yes	Yes	Yes	Yes
Time	Yes	Yes	Yes	Yes	Yes

Note

* * *, * *, * are significant at the level of 1%, 5% and 10%, respectively; t-statistics in parentheses.

### 5.3 Robustness test

#### 5.3.1 Adding control variables

This paper further adds cost of living (COL), foreign direct investment (FDI), and social consumption level (SCL) as control variables on the basis of the benchmark regression model. Among them, the cost of living is measured using the per capita consumption expenditure (including housing expenditure) of urban households as a share of disposable income, the amount of foreign direct investment is measured using the share of foreign direct investment in total regional GDP, and the level of social consumption is measured by the ratio of total retail sales of consumer goods to regional GDP. The specific regression results are shown in column (1) of Table 8. The development of the digital economy is still able to significantly increase employment polarisation, suggesting that the benchmark regression results are relatively robust.

**Table 8:** Robustness tests.

Variable	(1)	(2)	(3)	(4)
	EMP	EMP	DED	EMP
IV			0.176***	
			(0.005)	
L.DED		0.112***		
		(0.039)		
DED	0.073**			1.513***
	(0.030)			(0.389)
COL	0.045			
	(0.047)			
FDI	-0.338**			
	(0.141)			
SCL	-0.112***			
	(0.030)			
_Cons	-0.234**	-0.221*		1.237***
	(0.106)	(0.117)		(0.357)
Other control variables	Yes	Yes	Yes	Yes
Individual	Yes	Yes	Yes	Yes
Time	Yes	Yes	Yes	Yes
Phase I F statistics			13.07	
Cragg-Donald Wald F statistic				8.84
Kleibergen-Paap rk LM_P statistic				12.97***

Note

***, ** and * are significant at 1%, 5% and 10% levels, respectively. Standard error is reported in parentheses.

#### 5.3.2 Replacing explanatory variables

Column (2) in Table 8 shows the estimation results using the level of digital economy development in the lagged period as an explanatory variable, and the coefficient of the impact of the level of digital economy development in the lagged period on employment polarisation is significantly positive. It is consistent with the findings of this paper.

#### 5.3.3 Considering endogeneity

Considering the problem of multiple endogeneity due to the possible existence of certain omitted variables in the model, data measurement error terms, model setup, etc., this paper adopts the two-stage least squares (2SLS) method to conduct endogeneity tests, with reference to the treatment of Zhao et al. [63], where the number of landline telephones in 1984 is logarithmically multiplied by the constructed cross-multiplier term of the number of households as the final instrumental variable to be used, and the results obtained are shown in Table 8. Columns (3)-(4) show that the robustness of the benchmark regression is confirmed.

## 6 Conclusions and policy recommendations

### 6.1 Research conclusion

This article is based on the theoretical framework of the digital economy and constructs four dimensions including digital foundation, application, innovation, and benefits. The entropy weight method is used to quantitatively analyze the development level of the digital economy in China’s provincial panel data from 2010 to 2020. This study combines a configuration perspective and a contingency perspective, using fsQCA and econometric regression models to explore various factors and their configuration paths that affect the polarization of labor employment structure. The study draws the following conclusions:

1. Labor force employment polarization is not caused by a single factor, but is subject to the interaction of multiple factors centered on the digital economy. The study found that there are four main configuration paths, namely digital society environment driven, digital education environment driven, digital economy environment driven, and other factor driven. Among them, the coverage of digital society driven environments is the highest, followed by digital education driven environments, while the coverage of digital economy driven environments and other factor driven environments is relatively low.

2. Overall, the digital economy, urbanization level, human capital level, and education expenditure have a significant promoting effect on employment polarization, while industrial agglomeration helps to alleviate the formation of employment polarization.

3. Compared with the influence of a single net factor, the combination effect of the configuration path can more significantly promote the formation of employment polarization, indicating that the systematic effect generated by the interaction between factors is greater than the sum of parts. This discovery validates the effectiveness of fsQCA in identifying configuration paths in case studies.

### 6.2 Policy suggestion

Based on the conclusions of this study, the article proposes the following suggestions:

Firstly, accelerate digital transformation and promote the deep integration of the digital economy and the real economy. Encourage enterprises to actively adopt digital technology and Internet platforms to improve productivity and innovation. At the same time, we should accelerate the construction of emerging industries and business models with high-tech industries as the core, expand the creative effects of the digital economy, increase employment opportunities, and meet the needs of the job market.

Secondly, the government should strengthen social security and welfare for the elderly service industry to meet the job demands of workers with different skill levels and enhance the flexibility of the labor market. In addition, efforts should be made to increase urbanization construction, support innovation and entrepreneurship activities through measures such as reducing taxes and fees, increasing entrepreneurship subsidies, etc., establish a more complete social public system, reduce employment risks, and ensure that workers enjoy fair employment opportunities.

Thirdly, in view of the urgent demand for high-tech talents in the digital economy industry, it is necessary to continuously increase education investment, promote close cooperation between higher education institutions and enterprises, integrate new technologies and professional skills into traditional subject education, and build a diversified education system to cultivate high skilled talents that meet the needs of economic and social development.

Fourthly, enhance the employment competitiveness of high and low skilled workers. Given technological progress and the high demand for high salaries in high skilled positions, the government should strengthen training mechanisms to help the workforce learn new skills and achieve job transfers to high skilled positions. At the same time, the government should provide entrepreneurship training and investment support, encourage low skilled workers to become self-employed or entrepreneurial, thereby creating more employment opportunities and improving the income and economic independence of low skilled workers. Fifthly, the establishment of a cross-sectoral coordination mechanism to formulate a unified policy framework and coordinate the allocation of financial resources can avoid wasted resources and ensure the effective use of funds. In addition, strengthening cooperation with local governments and social organisations to increase social participation and public awareness of policies is also key to achieving policy objectives.

Finally, the establishment of feedback and monitoring mechanisms, as well as the fostering of a culture of cross-sectoral cooperation, will further enhance the transparency and accountability of policy implementation, ensuring that policies can truly meet the needs of the labour market and promote the healthy development of the job market.

## 7 Research limitations and perspectives

This study has achieved some results in exploring the driving factors, configuration paths and their effects of employment polarization under the development of digital economy, but there are still some limitations.

First, although economic, social and educational factors are selected, the model may not cover all the factors that may affect employment polarization, such as regional cultural differences and labor mobility. Second, the study mainly focuses on the direct impact of the digital economy on employment polarization, while the indirect effects and potential long-term impacts are insufficiently explored. Finally, the availability of data limits the in-depth understanding of the phenomenon of employment polarization in a broader spatio-temporal context.

To address the above limitations, future research can expand and deepen in the following areas. First, consider introducing more influencing factors, such as labor mobility and regional cultural differences, in order to construct a more comprehensive analytical framework for employment polarization. Second, with the continuous advancement of digital technology, future research can focus on the potential impact of emerging technologies, such as artificial intelligence and big data, on employment polarization, and how to respond to these changes through education and training policies to promote the healthy development of the labor market. Finally, factors affecting the employment structure are influenced by multiple factors, including the employment preferences of the employed, the pressure of the social environment and personal factors, and it is hoped that future research will take into account the impact of micro-level factors on the employment structure.
